# Exploring the relationships between visuospatial working memory, math, letter-sound knowledge, motor competence, and gender in first grade children: A correlational study

**DOI:** 10.3389/fpsyg.2022.981915

**Published:** 2023-01-19

**Authors:** Adrian Dybfest Eriksen, Alexander Olsen, Hermundur Sigmundsson

**Affiliations:** ^1^Department of Psychology, Norwegian University of Science and Technology, Trondheim, Norway; ^2^Department of Physical Medicine and Rehabilitation, St Olavs Hospital, Trondheim University Hospital, Trondheim, Norway; ^3^Reykjavik University, Reykjavik, Iceland

**Keywords:** skill correlations, reading, arithmetic, executive functions, cognitive control, motor skills, sex differences, first grade development

## Abstract

**Introduction:**

Development of crucial skills accelerates at the start of formal schooling, although, more knowledge is needed about the relationships between such skills. The current study explored the relationships between visuospatial working memory, letter-sound knowledge, math competence and motor competence, as well as potential effects of gender.

**Materials and methods:**

The sample consisted of 85 (42 girls) 6 to 7 years old first grade children, and was measured with a test battery consisting of tests designed for each skill domain.

**Results:**

Results demonstrated weak to moderate statistically significant correlations between visuospatial working memory, letter-sound knowledge, math competence, with no statistically significant gender differences. Two motor tasks measuring manual dexterity, placing bricks and building bricks, showed a weak statistically significant correlation.

**Discussion:**

We argue that the findings demonstrate the relationships between these skills are low to moderate in first grade. Furthermore, we argue that these skills ought to be trained deliberately. The potential role of visuospatial working memory in procurement of novel skills in early childhood ought to be explored further in future studies.

## Introduction

In this study, the relationships between visuospatial working memory (VSWM), math skills, letter-sound knowledge (LSK), motor competence, and gender differences in first grade children are examined. A multitude of cognitive skills seems to develop in tandem with academic skills in early child development. However, there is a call for further exploration of potential interrelationships among such skills, especially regarding cognitive skills in early formal schooling (Purpura and Schmitt, 2019). According to Fleishman a skill is “the level of proficiency on a specific task or limited group of tasks” (Fleishman, 1966, p. 148). A skill could therefore include both behavior and knowledge. The nature of the relationships between skills could be explained by principles of learning and skill development which states that different neural networks are involved in different tasks and therefore require specific training (Edelman, 1993; Kleim and Jones, 2008; Sigmundsson et al., 2017b). It is argued that strong and consistent correlations between tasks could indicate that an underlying factor (i.e., ability) influences the performances in those tasks. On the other hand, weak correlations might indicate skill specificity. To our knowledge, no studies have explored the relationships between VSWM, math skills, LSK and motor competence in conjunction. The same is true for gender differences in these relationships. The present study’s primary aim is to explore the relationships between skills in first grade children. The secondary aim is to explore potential gender differences in the relationships. The following sections aim to present an overview of literature on how the different skills are related and whether these relationships differ between genders.

The capacity to retain visual and spatial information is important for everyday life and academic success. For example, retaining the numerals in a math equation or keeping one’s attention on letters and words in a sentence. VSWM is the capacity to actively retain visual and spatial information for a short duration of time (Baddeley, 1996) and draws on cognitive control functions such as attention and inhibition (Miyake et al., 2001; Salminen et al., 2012; Diamond, 2013). In this regard, VSWM seems to be an important component for learning new skills (Andersson, 2008; Gathercole et al., 2019) and has in many studies been linked with early academic performance (Bull and Scerif, 2001; Alloway et al., 2006; St Clair-Thompson and Gathercole, 2006; McClelland et al., 2007; Bull and Lee, 2014; Zelazo et al., 2016; Allen et al., 2019). For example, one study of 6–9th grade students revealed that VSWM was moderately associated with math competence, motor skills, and several other measures of cognitive functioning (Syväoja et al., 2021). Ashkenazi et al. (2013) and De Smedt et al. (2009) both described VSWM as a source of domain general vulnerability in early development of math skills in both normal and abnormal populations. This could partly be explained by the shared neural circuits for VSWM and mathematics (Matejko and Ansari, 2021). Moreover, Bourke et al. (2014) found that VSWM uniquely contributed to the ability to transcribe upper and lower case letters of the alphabet, which the authors explained as a function of converting ideas and language into meaningful visual units. Even though VSWM is considered a domain general capacity, a meta-analytic review revealed that VSWM training has a limited, short-term effect on other skills domains, such as reading and arithmetic (Melby-Lervåg et al., 2016). The highest transfer effect seen in this meta study was found between tasks with high similarity. Considering the different findings presented here, it is interesting to further explore how VSWM correlates with other important skills. In sum, VSWM might be considered a specific skill at the behavioral level which can be improved upon by training, in addition to being a general capacity that seems to influence the ability to perform well in novel skills.

Reading is one of the most crucial skills to attain in early childhood. It enables us to gather knowledge from text and participate in society. The simple view of reading pertains two important factors in learning to read: decoding and language comprehension (Hjetland et al., 2019; Nation, 2019). It may be argued that LSK, i.e., connecting graphemes with phonemes, is of crucial importance at the beginning of formal schooling (Ehri, 2005; Dehaene, 2011; Tønnessen and Uppstad, 2015). LSK is linked to the visual word form area (VWFA) in the fusiform gyrus, is highly experience dependent, and therefore requires specific practice (Dehaene et al., 2015; Nation, 2019). Furthermore, Chen et al. (2019) found that the VWFA shows neural connections to both language and visuo-spatial attention circuits which could partly explain the connection between VSWM and learning to read. Even though learning to read is a highly specific process (Nation, 2019), reading comprehension has been linked to math competence in several studies (Purpura et al., 2011; Sánchez-Barrioluengo and Soto-Calvo, 2011; Korpipää et al., 2017; Balhinez and Shaul, 2019). To summarize the findings from these studies, the relationships are reported as moderate. The association between reading and math could be a result of reading’s general importance for children’s academic development (Rose, 2006; Dehaene, 2011) and perhaps the shared underlying cognitive processes of math and reading, such as VSWM (Ashkenazi et al., 2013; Bourke et al., 2014; Chen et al., 2019). In the current study we expect moderate correlations between math competence and LSK, and between VSWM and LSK.

Attaining basic competence in mathematics from an early age is important for academic success. Math competence is a composite of basic number knowledge and skills for manipulating sets of numbers through specific operations (Butterworth, 2005; Dehaene, 2008). The development of math skills, seems to rely on a “number sense,” i.e., an innate knowledge about quantities (Geary and Bjorklund, 2000; Dehaene, 2004; Butterworth, 2005), and deliberate training of specific arithmetic operations (Butterworth, 2005; Sigmundsson et al., 2013). As previously mentioned, there seems to be a distinct relationship between VSWM and performance in math skills in early schooling. For example, Fanari et al. (2019) found that at the beginning of first grade, performance in math was predicted by spatial working memory, while at the end of second grade both visual and spatial working memory predicted math competence explaining about 25% of the total variance. VSWM is thought to affect math skills by retaining and manipulating the intermediate steps in finding a solution to mathematical problems (Peng et al., 2015). In addition, VSWM and math tasks show common neural substrates during childhood (Matejko and Ansari, 2021). A recent systematic review by Allen et al. (2019) found an overall positive relationship between math performance and VSWM in children at the behavioral level. VSWM has positive associations with several types of math skills, although more so with subtraction and addition in early grades (Van Der Ven et al., 2013). Findings also suggest that at different levels of skill acquisition, working memory is more involved in procuring novel skills, that is, before the children are able to retrieve well-practiced strategies from long-term memory (Bull and Scerif, 2001; Andersson, 2008; Gathercole et al., 2019). Based on the current literature, we expect a moderate to strong relationship between VSWM and math competence in early schooling.

The fourth skill domain explored in this study is motor competence. Motor competence is a sum composite of fine and gross motor skills (Sigmundsson et al., 2016). Fine motor skills are movements involving small muscle groups such as hands and fingers, while gross motor skills include larger movements of big muscle groups—often in tandem with balance. Developing such skills are important for everyday functioning. There is some evidence pointing to the co-development of motor skills and VSWM (Seidler et al., 2012; Stöckel and Hughes, 2016; McClelland and Cameron, 2019), as well math and reading (Pitchford et al., 2016; McClelland and Cameron, 2019). However, these studies do not claim a causal link between the skills, rather, that executive functions and motor skills are dynamically dependent in processes like error correction in motor control and provides a basis for classroom learning, e.g., sitting at a desk and reading, writing, and listening. Furthermore, different motor skills seem to be weakly correlated (Drowatzky and Zuccato, 1967; Haga et al., 2008). A recent study by Sigmundsson et al. (2021) showed that motor skill learning in 7–8-year-old is supported by the specificity argument. The specificity of motor skills is further supported by the principles of Fleishman’s differential approach to skill development (Fleishman, 1975) and the neurobiological underpinnings of motor skill development (Sporns and Edelman, 1993). It is therefore reasonable to expect low correlations between different motor tasks and between motor competence and other skills in the present study.

Gender differences in academic skills is a widely studied topic and the research has many implications for how schools and tutors ought to implement teaching strategies that works for either gender. However, there seems to be a knowledge gap in research on gender differences in the relationships between VSWM, LSK, math competence, and motor competence in early formal schooling. In the present paper, we therefore aim to explore any potential gender differences in the relationships themselves. Investigating gender differences and similarities in the relationships themselves could further help elaborate how boys and girls learn different skills from an early age.

In the current study, the measurements of math competence and LSK are both derived from the Norwegian curriculum, although how this affects the relationships between the skills is uncertain. In this context, Norway is especially interesting as gender differences in reading are one of the most prominent both nationally (Sigmundsson et al., 2017a, 2018) and internationally (Borgonovi et al., 2018b; OECD, 2019). On the other hand, performance in math is relatively equal according to large-scale studies (Borgonovi et al., 2018b; OECD, 2019). According to one study females generally perform better at curriculum-based measurements in math throughout elementary school (Yarbrough et al., 2017). Gender differences in math and reading are also found to be inversely related, meaning that countries with higher gender equality show less differences in math and larger differences in reading and vice versa for countries with lower gender equality (Stoet and Geary, 2013). Furthermore, males tend to show larger variability in skill measures compared to girls (Hyde, 2014).

It is worth noting that gender differences in motor skills are an understudied phenomenon where most of the research was carried out in the past century (Liutsko et al., 2020). Updated literature regarding gender differences in the relationships between different motor skills and between motor competence and cognitive skills is therefore sparse. Only a few studies have found statistically significant differences in motor skill performance in children in recent times, e.g., female superiority in manual dexterity skills (Junaid and Fellowes, 2006; Flatters et al., 2014; Mathisen, 2016) and selected measures of balance (Sigmundsson and Rostoft, 2003).

Very few studies have investigated the links between gender, VSWM, math, and reading. The studies that do exist were carried out on adult populations. For example, Paz-Baruch (2022) found no gender differences in the associations between VSWM, math, and reading performance in a high-school cohort. For first grade children no such data exist, although some studies suggest that gender has no influence on VSWM performance in first grade (Alloway et al., 2006; Voyer et al., 2007). A gender effect in VSWM has been observed from adolescence, where girls display better object location memory compared with boys (Voyer et al., 2017) indicating that age is of relevance here. The links between VSWM and other skills in children need to be researched.

Gender could have potential effects on the relationships between the different skills addressed in this study, although, the literature on this phenomenon is lacking. In sum, we can expect that girls might perform better than boys in LSK and therefore display stronger relationships between LSK and other skills. Since many math tasks involve reading skills and underlying VSWM-processes, it is reasonable to expect that the relationships between VSWM, reading, and maths are stronger for girls than for boys. We expect overall low correlations between different kinds of motor skills and between motor competence and other skills, regardless of gender.

In sum, VSWM is associated with performance in math (De Smedt et al., 2009; Ashkenazi et al., 2013), reading skills (Bourke et al., 2014), and motor skills (McClelland and Cameron, 2019), arguably as an underlying influence in the procurement of novel skills (Bull and Scerif, 2001; Andersson, 2008; Gathercole et al., 2019). Math and reading are reported as moderately to strongly associated in the literature (e.g., Korpipää et al., 2017; Balhinez and Shaul, 2019). Regarding motor competence, the literature indicates a high degree of specificity between different motor skills (e.g., Sigmundsson et al., 2021). As for gender differences in the relationships between skills, the literature is lacking. Although, girls seem to perform better in reading from an early age (e.g., Sigmundsson et al., 2018; Borgonovi et al., 2018a) and object location memory from adolescence (Voyer et al., 2007). We expect stronger relationships between skills for girls compared with boys. Based on previous findings, one could expect multicollinearity with moderate to strong associations between most variables, except motor competence. Consistently strong relationships between factors could indicate underlying abilities (Fleishman, 1975; Kleim and Jones, 2008; Sigmundsson et al., 2017b). The primary aim of this study is to explore the relationships between VSWM, LSK, math competence, and motor competence, in first grade children. The secondary aim is to investigate potential gender differences in the relationships.

## Materials and methods

### Study design and participants

The sample consists of 85 children, between the ages of 6–7 [43 males and 42 females, mean age = 6.77 years (81.22 months), age SD = 3.15, range = 75–86 months]. Mean age for males was 6.72 years (80.70 months, SD = 3.14, range = 75–86 months). Mean age for females was 6.81 years (81.71 months, SD = 3.17, range = 75–86 months). The children were recruited from a representative elementary school in Norway. As part of the inclusion criteria, none of the participants had any diagnosed learning disorders or any other medical conditions that could interfere with test performance. The project was approved by Regional Committees for Medical and Health Research Ethics of Norway (REC) and all participants had written and informed consent from parents in accordance with the Helsinki Declaration.

### Measurements and procedure

#### Visuospatial working memory

The computer-based test Spanboard (Westerberg et al., 2004) was used in assessment of VSWM. The test is a dot-matrix task aimed to measure the accuracy and span of object location memory. Memory stimuli were presented as red disks (circles) in a four-by-four grid on a laptop computer screen 35–40 cm from the face. The participant’s task was to indicate the red circles’ location in an empty grid. After all stimuli were presented in each trial, response was to be made. Each participant had a trial run consisting of two trials with two circles to ensure correct execution. The number of circles increased chronologically after every second trial, ranging from two to nine circles. When accomplishment of both trials was not successful, the test terminated, and the scores were logged. Test score was the correct number of clicks which represents the accuracy of retention and the span of VSWM (Westerberg et al., 2004). The grid on screen was 22 cm × 22 cm and each circle was visible on screen for 2,250 ms, and the time between each stimulus (inter-stimuli interval) was 750 ms. Test duration was approximately 5–10 min.

#### Letter-sound knowledge

In order to assess the participants’ knowledge of the graphemes and phonemes of the alphabet, the Letter-Sound Knowledge Test (LSK test; Ofteland, 1992) was used. One letter at a time was presented visually, in a non-alphabetic order. The participants were then asked to:

1. Indicate how many of the uppercase letters of alphabet they know (A, B, C, etc.) by providing the name the letters they know.
2. Demonstrate their knowledge of the respective sounds of the uppercase letters.
3. Indicate how many of the lowercase letters of the alphabet they know (a, b, c, etc.) by providing the name the letters they know.
4. Demonstrate their knowledge of the respective sounds of the lowercase letters.

The Norwegian alphabet consists of 29 letters and is considered a semi-transparent orthography. The test consists of two sheets of paper, one for uppercase and one for lowercase letters. Participants were seated in a chair next to a table of suitable height for the testing. The LSK-test has been shown to have high construct validity (Spearman’s Rho = 0.683) and test–retest reliability with an intra-class correlation of 0.985–0.992 (see Ofteland, 1992; Sigmundsson et al., 2018). Test duration is roughly 10–15 min.

#### Math competence

In order to assess math competence the Norwegian version of the Think Mathematically!-test [Alle Teller!-test (TM-test)] developed by McIntosh et al. (2007) was used. The test was developed in collaboration with the Norwegian Center for Mathematics Education and is based on the Norwegian elementary and middle school curriculum, from first to tenth grade, as a tool for teachers to chart individual students’ skill level (McIntosh et al., 2007). The test for first graders includes 15 tasks regarding basic addition, subtraction, and concepts numerosity. Administration was done digitally on a laptop computer, where the participant was seated in a suitable chair with the screen approximately 35–40 cm from the face. The experimenter read the questions out loud and made sure the participant understood the tasks; and the participant was then instructed to indicate what s/he considered the correct answer. Some tasks required only verbal responses, other tasks required pointing at and clicking on the screen. The experimenter was instructed not to give away any correct answers, but instead encourage a response from the participant. The test takes roughly 10–15 min to complete. Higher score (0–15) indicates higher competence. Average inner consistency (Cronbach’s alpha) for the TM-test range from 0.75 to 0.92 (Reitan and Imsen, 2012).

#### Motor competence

The Test of Motor Competence (TMC; Sigmundsson et al., 2016) was used to assess motor competence. The participant is required to perform each motor task in a specified manner for valid results. Two tests for manual dexterity: Placing bricks and building bricks; and two for dynamic balance: Walking/running in slopes (W/R) and heel to toe walking (HTW). For the Placing Bricks task (PB), the participant is instructed to place 18 square-shaped bricks on a board (Duplo™) in the fastest possible manner. The bricks are placed in horizontal lines of three on the side of the active hand, while the other hand holds the board firmly. Both hands are tested sequentially. For the Building Bricks test (BB), the task is to build a tower of 12 Duplo™ bricks. Before the start signal, the participant is required to hold one brick in each hand, while the arms are to be held in the air and not touch the table for the duration of the task. The task itself then consists of assembling the tower, stacking one, and one brick together. During the PB and BB tasks, the participant is seated comfortably at a table of suitable height. HTW requires the participant to walk down a 4.5-meter-long straight line (marked on the floor with tape) placing the heel against the toes of the foot in each step. For the W/R task, the participant runs or walks from the starting point in a figure of eight around two marked lines (1 m in width). Line 1 is 1 m from the starting line, and Line 2 is 5.5 m from the starting line. The figure of eight is accomplished by stepping to the left or right of Line 1 and go back on opposite side (right or left side) of Line 2. Suitable footwear was worn. All the tests are measured in time; whereof lower scores indicate higher motor performance. The standardized TMC is designed for testing motor competence in a lifespan and is a widely used quantitative tool for assessing motor skills. The test battery has a test–retest reliability of 0.87 (Sigmundsson et al., 2016).

### Procedure

The children were tested at one time-point during the months of March/April in a suitable quiet room at their school during school hours. These months were chosen due to the completion of alphabet tuition at this school. The first author (ADE) and three trained research assistants carried out all tests according to test protocol. Each child was tested individually, and test order was randomized by allotting different starting locations and an ID-number. Participants were verbally encouraged and supported during the procedures, and if any procedural errors occurred, instructions and demonstrations were repeated and a retry was initiated.

### Analysis

SPSS version 24 for Windows was used for analysis (SPSS, Inc., Chicago, IL, United States). Descriptives for each measure were calculated and are shown in Table 1. Descriptives include medians, interquartile ranges (the differences between the 75 and 25th percentile), means, and SDs. For analyses of the relationships between the variables, the non-parametric Spearman’s Rho test was used in this study. The decision was made due to violations of assumptions for parametric testing, as determined by investigating histograms with normal distributions which revealed a negative skewness with possible ceiling effect for LSK and math competence; test of normality (Kolmogorov–Smirnov with Lilliefors significance correction); and a test of homogeneity of variance (Levene’s test) on all variables. Additionally, the sample size of 85 (boys *n* = 43) is a relatively moderate sample size further warranting non-parametric analyses.

**Table 1:** Descriptive statistics of LSK, math competence, VSWM, and motor competence in first graders.

	Median	IQR	Mean	SD	N
Average LSK	26.25	24–27.75	25.01	3.98	85
Uppercase letter names	28	25–29	26.2	4.14	85
Uppercase letter sounds	26	24–27.50	25.02	3.95	85
Lowercase letter names	27	24–28	24.88	4.65	85
Lowercase letter sounds	25	23–27	23.92	4.21	85
Math	13	12–14	12.89	1.66	85
VSWM	17	12–22	16.92	6.15	85
Motor competence	−0.06	−0.50–0.48	0	0.57	85
WRS	13.6	10.45–15.98	14.1	5.02	85
HTW	28.2	19.22–42.09	31.43	15.2	85
BB	30	24.33–40.78	33.71	14.17	85
PB	40.7	34.49–47.78	40.78	8.3	85

Interquartile range (IQR) is the 25th to the 75th percentile. LSK, letter-sound knowledge; VSWM, visuospatial working memory; WRS, walking/running in slopes; HTW, heel to toe walking; BB, building bricks; and PB, placing bricks.

Spearman’s Rho test is a rank-order probability analysis of covariance. The variables’ scores are ranked, and the means of the ranks are then then tested for covariance with the other variables’ mean ranks. The null-hypothesis for a Spearman’s Rho test states that the relationship equals zero, i.e., as the mean ranks of one variable increase, the mean ranks of another variable do not increase or decrease. The rule for intepreting the coefficients is that *r*_s_ < 0.30 is considered weak, *r*_s_ between 0.30 and 0.80 is moderate, and *r*_s_ > 0.80 indicate a strong relationship (Kraska-Miller, 2019). In this context, the median scores for the whole group are compared, followed by individual analyses for boys and girls separately. Gender was coded 0 for boys and 1 for girls. Age is the number of months at the time of testing (March/April). Data transformation was used for two variables: average letter score, which is the average of big and small letter name and sound, with a maximum score of 29, e.g., $\frac{\left(29+29+29+29\right)}{4}$; and motor competence where the four subtests were transformed into one variable using standardized *z*-values (see Sigmundsson et al., 2016). None of the other variables in the test battery were transformed.

## Results

In Table 1, descriptive statistics for LSK, math competence, VSWM and motor competence, as well as the subcategories of LSK and motor competence, are displayed. Tables 2, 3 display descriptive statistics for males and females separately.

**Table 2:** Descriptive statistics of LSK, math competence, VSWM, and motor competence in first graders for males.

	Median	IQR	Mean	SD	*N*
Average LSK	26	23.25–27.50	24.42	4.39	43
Uppercase letter names	27	25–29	25.56	4.64	43
Uppercase letter sounds	26	23–27	24.72	4.33	43
Lowercase letter names	26	23–27	24.14	5.05	43
Lowercase letter sounds	25	22–28	23.26	4.56	43
Math	13	12–14	12.84	1.7	43
VSWM	17	11–22	16.6	6.23	43
Motor competence	−0.05	−0.45–0.32	0	0.6	43
WRS	13.99	10.42–18.90	14.77	5.75	43
HTW	28.89	19.32–43.66	31.29	14.21	43
BB	30.14	24.27–35.75	32.74	12.76	43
PB	38.45	34.13–47.95	40.28	8.75	43

Interquartile range (IQR) is the 25th to the 75th percentile. LSK, letter-sound knowledge; VSWM, visuospatial working memory; WRS, walking/running in slopes; HTW, heel to toe walking; BB, building bricks; and PB, placing bricks.

**Table 3:** Descriptive statistics of LSK, math competence, VSWM, and motor competence in first graders for females.

	Median	IQR	Mean	SD	*N*
Average LSK	26.5	25.19–27.81	25.61	3.45	42
Uppercase letter names	28	27–29	26.86	3.5	42
Uppercase letter sounds	26	24.75–28	25.33	3.56	42
Lowercase letter names	27	25–28	25.64	4.14	42
Lowercase letter sounds	25	24–27	24.6	3.75	42
Math	13	12–14	12.95	1.64	42
VSWM	17.5	12.75–21.25	17.24	6.09	42
Motor competence	−0.05	−0.41–0.36	−0.01	0.55	42
WRS	13.5	10.62–14.72	13.4	4.1	42
HTW	27.52	18.77–39.94	31.57	16.32	42
BB	29.98	24.04–41.89	34.04	14.97	42
PB	41.3	34.63–47.16	41.3	7.9	42

Interquartile range (IQR) is the 25th to the 75th percentile. LSK, letter-sound knowledge; VSWM, visuospatial working memory; WRS, walking/running in slopes; HTW, heel to toe walking; BB, building bricks; and PB, placing bricks.

**Figure 1:**
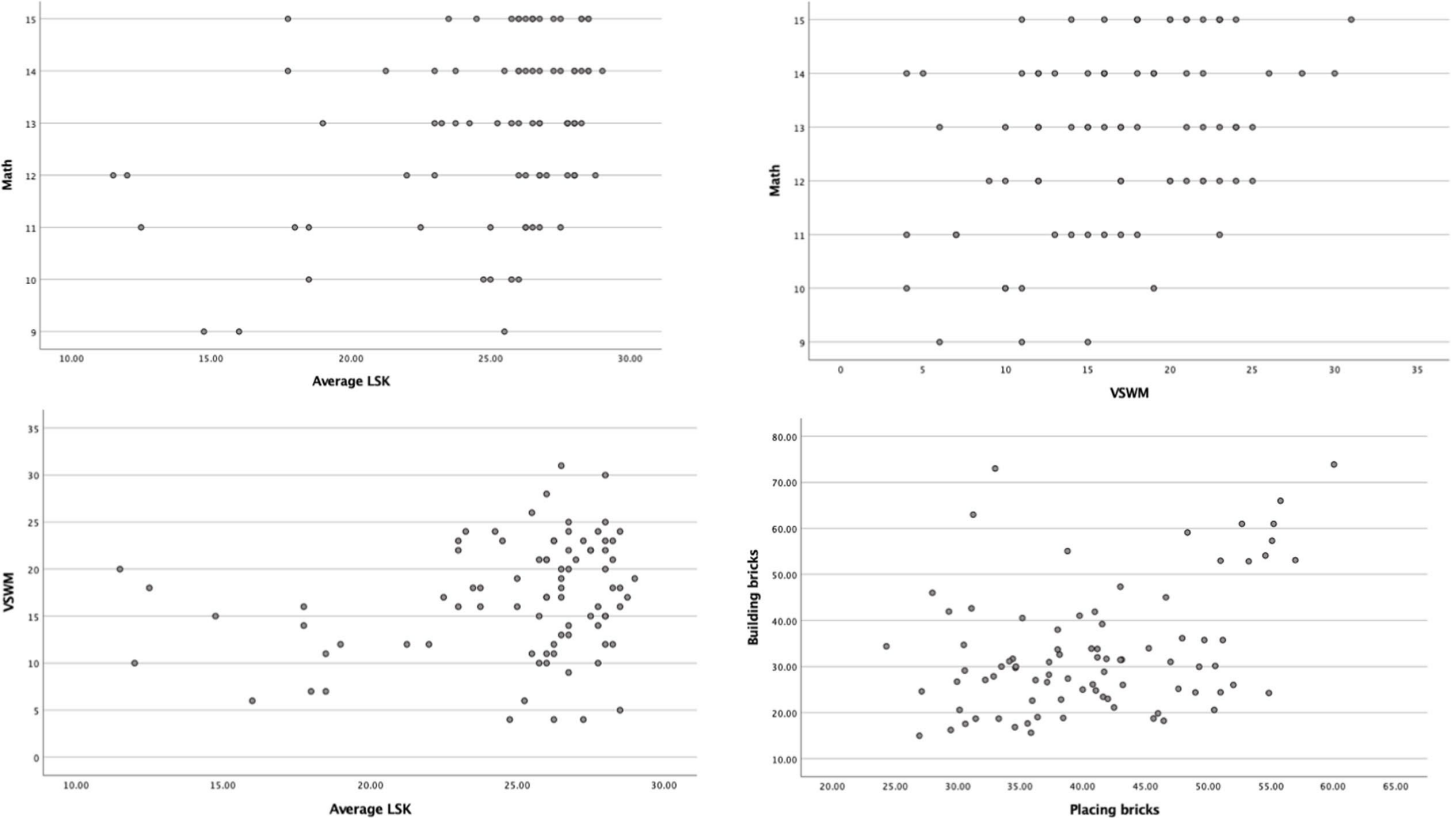
Scatterplots of the statistically significant correlations for the whole group.

Relationships between average LSK, math competence, VSWM, and total score on motor competence for the whole sample (*N* = 85) are shown in Table 4. In total, the scores for VSWM were moderately correlated with math competence (*r*_s_ = 0.355, *r*_s_^2^ = 0.126, *p* = 0.001), and weakly correlated with average LSK (*r*_s_ = 0.223, *r*_s_^2^ = 0.050, *p* = 0.040). Math competence was also moderately correlated with average LSK (*r*_s_ = 0.302, *r*_s_^2^ = 0.091, *p* = 0.005). The scores for motor competence showed no significant correlations with any other variable (Figure 1).

**Table 4:** Results from Spearman’s Rho bivariate correlation analysis (*N* = 85).

	VSWM	Average LSK	Math
Average LSK	0.223^*^		
Math	0.355^**^	0.302^*^	
TMC	0.103	0.169	0.171

LSK, letter-sound knowledge; VSWM, visuospatial working memory; and TMC, test of motor competence. ^*^p < 0.05, ^**^p < 0.01.

Tables 5, 6 represent the correlations for males (Table 5) and females (Table 6). Overall, the scores for VSWM tended to be moderately correlated to math competence for both genders, although somewhat stronger for females [*r*_s_(43) = 0.302, *r*_s_^2^ = 0.180, *p* = 0.005] compared to males [*r*_s_(42) = 0.321, *r*_s_^2^ = 0.103, *p* = 0.036]. For females, math competence also correlated with average LSK [*r*_s_(43) = 0.475, *r*_s_^2^ = 0.226, *p* = 0.001], which was the strongest significant correlation observed in this study. This trend was not displayed in the male cohort where average LSK showed no significant relationships with any other variable. Additionally, motor competence showed no relationships with any other variable for both genders. Fisher-Z-transformation of Spearman Rho showed no statistically significant differences between the correlations of males and females, as all *z* values were below the critical value 1.96 (*p* > 0.05).

**Table 5:** Results from Spearman’s Rho bivariate correlation analysis for males (*n* = 43).

	VSWM	Average LSK	Math
Average LSK	0.245		
Math	0.321^*^	0.150	
TMC	0.147	0.069	0.193

LSK, letter-sound knowledge; VSWM, visuospatial working memory; and TMC, test of motor competence.

^*^p < 0.05.

**Table 6:** Results from Spearman’s Rho bivariate correlation analysis for females (*n* = 42).

	VSWM	Average LSK	Math
Average LSK	0.199		
Math	0.422^*^	0.475^**^	
TMC	0.019	0.303	0.133

LSK, letter-sound knowledge; VSWM, visuospatial working memory; and TMC, test of motor competence.

^*^p < 0.05, ^**^p < 0.01.

Table 7 shows the relationships between the different motor skills, and Tables 8, 9 show the motor skill relationships for males and females, respectively. One statistically significant correlation was observed for the whole cohort, between BB and PB [*r*_s_(85) = 0.269, *r*_s_^2^ = 0.072, *p* = 0.013]. No statistically significant gender differences in the relationships were found (*p* > 0.05).

**Table 7:** Results from Spearman’s Rho bivariate correlation analysis for motor competence (*N* = 85).

	WRS	HTW	BB
HTW	−0.090		
BB	−0.038	0.081	
PB	0.150	0.199	0.288^*^

WRS, walking/running in slopes; HTW, heel to toe walking; BB, building bricks; and PB, Placing bricks.

^*^p < 0.05, ^**^p < 0.01.

**Table 8:** Results from Spearman’s Rho bivariate correlation analysis for motor competence for males (*n* = 43).

	WRS	HTW	BB
HTW	0.245		
BB	0.072	0.131	
PB	0.196	0.187	0.278

WRS, walking/running in slopes; HTW, heel to toe walking; BB, building bricks; and PB, placing bricks.

^*^p < 0.05, ^**^p < 0.01.

**Table 9:** Results from Spearman’s Rho bivariate correlation analysis for motor competence for females (*n* = 42).

	WRS	HTW	BB
HTW	0.070		
BB	−0.165	0.045	
PB	0.006	0.215	0.325^*^

WRS, walking/running in slopes; HTW, heel to toe walking; BB, building bricks; and PB, placing bricks.

^*^p < 0.05, ^**^p < 0.01.

## Discussion

The present study aimed to explore the relationship between visuospatial working memory (VSWM), letter-sound knowledge (LSK), math competence, motor competence, and potential gender differences. Results from the correlation analyses indicate weak to moderate statistically significant relationships between VSWM, math, and LSK for the whole cohort. These findings partly confirm our hypothesis of moderate relationships between VSWM, math, and LSK. One of the more striking findings in this study was the consistent pattern of moderate correlations between VSWM and math, which was evident in both females and males. On the other hand, VSWM showed no statistically significant association with LSK when investigating the genders separately. There was also no statistically significant correlation between math and LSK for males, but a moderate correlation was observed for females. However, there were no statistically significant gender differences in the relationships between skills, which contradict our hypothesis of females outperforming males in LSK. Additionally, motor competence showed no associations with other skills, and the intrarelationships of motor skills were generally low with one statistically significant correlation observed between the scores from the building bricks and placing bricks tasks, confirming our hypotheses regarding the relationships between motor skills and between motor competence and other skills.

Visuospatial working memory is frequently associated with mathematical skills (e.g., Andersson, 2008; Holmes et al., 2008; De Smedt et al., 2009; Ashkenazi et al., 2013; Li and Geary, 2013; Bull and Lee, 2014; Fanari et al., 2019) and early reading skills (e.g., Alloway, 2007; Bourke et al., 2014; Pham and Hasson, 2014). In contrast to prior studies, the relationships in the current study were not as strong, and motor competence was not related to VSWM. Calculation of *r*_s_-squared further elaborate the strengths of relationships and showed that VSWM share about 12.6, 5.6, and 4.9 percent of the rank variation in math competence, LSK and motor competence, respectively. In comparison, Fanari et al. (2019) found that VSWM explained about 25% of the total variance in math competence in first grade. The authors argue that VSWM involves both domain general and domain specific attributes, whose components could have differentiated impacts on mathematical skills in early schooling. This could partly be explained by the shared neural circuits of VSWM and math skills (Matejko and Ansari, 2021). Since VSWM is an integrative part of working memory and cognitive control (Bull and Scerif, 2001; Diamond, 2013; Gathercole et al., 2019) it could be that other latent variables, such as attention, inhibition, and verbal working memory, have an influence on the participants’ performance in math competence and LSK. This argument shows that VSWM is a part of a larger construct, i.e., cognitive control. As Melby-Lervåg et al. (2016) put it “…working memory training programs appear to produce short-term effects that do not generalize to tasks that have not been directly trained.” (pp. 513–514). This meta-study underlines that specific *training* of VSWM is not directly transferable to other skill domains, such as reading and math, but VSWM does seem to be an important part of a more domain general cognitive capacity (Baddeley, 2012; Diamond, 2013).

**Figure 2:**
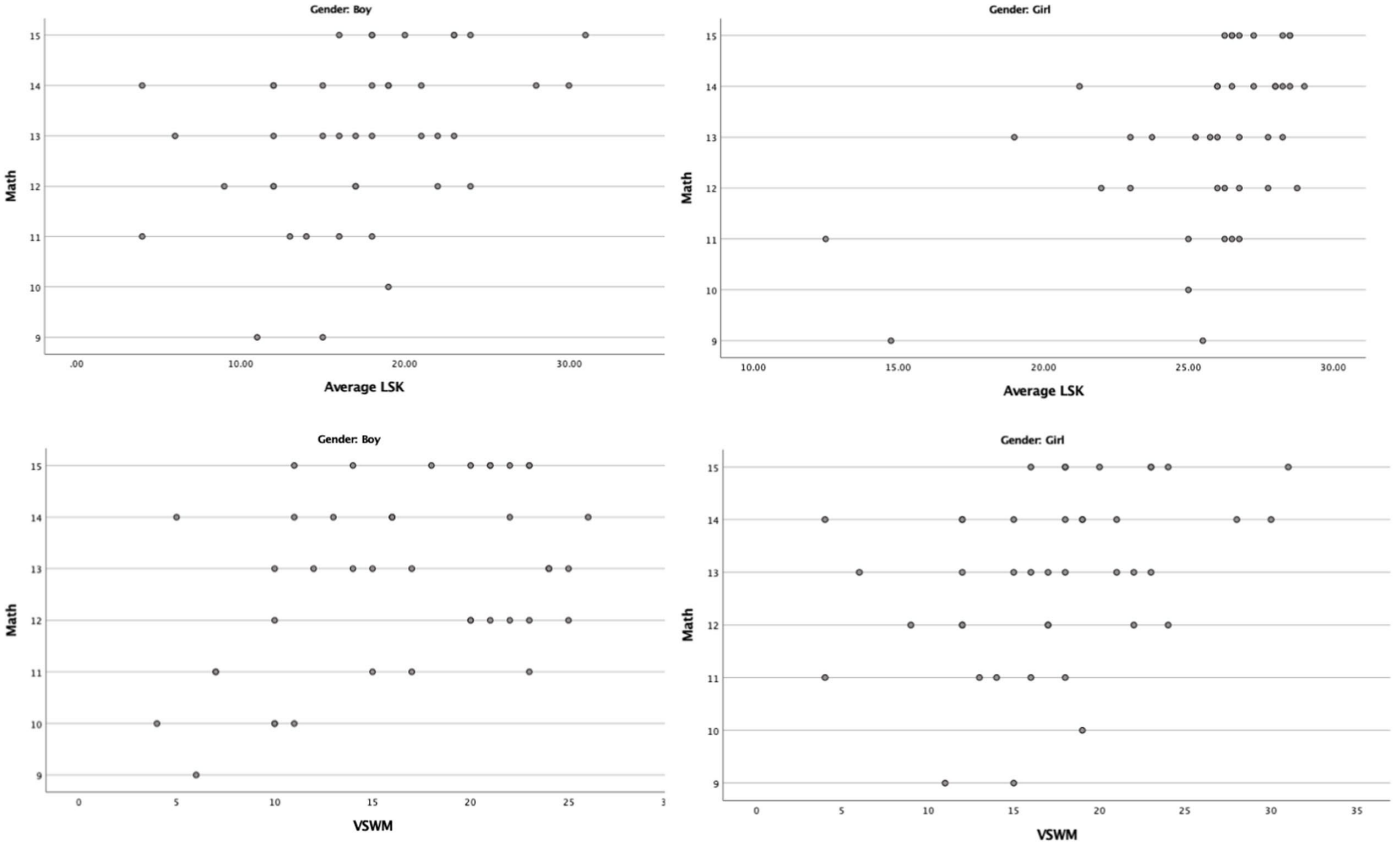
Scatterplots split by gender.

The strongest relationship (*r*_s_ = 0.475, *r*_s_^2^ = 0.226) was found between math and LSK for females. Associations between early reading skills and math competence have been demonstrated in previous studies (Purpura et al., 2011; Sánchez-Barrioluengo and Soto-Calvo, 2011; Korpipää et al., 2017; Balhinez and Shaul, 2019), although, to our knowledge, no studies have looked at potential gender differences in this association in first graders. There was no statistically significant relationship between math and LSK for males though, suggesting a possible gender nuance in the relationships between these skills. In curriculum-based measures, e.g., math and LSK, it has been shown that females somewhat outperform males arguably due to males being more varied in performance, i.e., the greater male variation hypothesis (Hyde, 2014) and the strict set of procedures and the language-strategies required to solve the tasks (Hyde et al., 1990; Halpern et al., 2007; Lindberg et al., 2010; Yarbrough et al., 2017). Figure 2 demonstrates that males show a less consistent pattern of relationship, while females show a stronger monotonic relationship, which might indicate that females have a more stable performance in both domains. However, no statistically significant differences were observed, as determined by Fisher-Z-transformation, and the findings should therefore not be considered evidence for a real-world effect of gender. Furthermore, the low and moderate correlations demonstrate that the skill relationships are mostly similar for both males and females in first grade.

Motor competence did not display any statistically significant relationships with other skills. In previous studies, motor competence have been found to have a positive relationship with some measures of VSWM in young children (Seidler et al., 2012; Stöckel and Hughes, 2016; McClelland and Cameron, 2019), likely due to the importance of the oculomotor system in error corrections and movement predictions (Seidler et al., 2012). Some studies even claim that motor competence is linked to both math and reading skills in early childhood (e.g., Pitchford et al., 2016). However, from the current findings it could be argued that there is a clearer divide between specific cognitive skills (i.e., VSWM, math and LSK) and motor skills, and that motor skills are more domain specific (Sigmundsson et al., 2021).

Indeed, upon further analysis of associations between the motor skills, placing bricks and Building bricks displayed a weak and statistically significant correlation. Similar findings have been found previously as well (e.g., Sigmundsson et al., 2016). This might be due to the similarity of the two tasks as they both measure manual dexterity and are somewhat similar in execution. The current findings demonstrate that even when two motor tasks are somewhat similar, the correlation is still found to be low. Furthermore, there seem to be large individual variations in the motor skills (see Table 1) which further suggests that motor skills are highly experience dependent (Sigmundsson et al., 2016). The results from the current study lend further support to this notion.

There are some limitations to this study that need further elaboration. Firstly, the sample is of a moderate size and was drawn from one school which limits generalizations from the findings. The study design was inspired by designs of similar studies which had 67 (Vedul-Kjelsås et al., 2012) and 73 (Sigmundsson et al., 2010) participants. Furthermore, appropriate analyses were utilized to tackle issues regarding moderately sized samples, non-normal and positively skewed distribution, as well as possible ceiling effects of math and LSK. See Figures 1, 2 for graphical representations of the relationships. No inferential conclusions regarding causality can be drawn from the results of a Spearman Rho correlation. There are also the issues of possible interaction effects between the variables and the “third variable”-problem, which correlation analyses cannot discern. Although, the aim of the current study was to explore the relationship between the variables, and as such Spearman Rho is a sensitive measure of how the participants are ranked and therefore tells us the strength of relationships (*r*_s_) and the proportion of shared variation (*r*_s_^2^) between two ranked variables (Kraska-Miller, 2019). Some of the variables are composites, such as math competence and motor competence, which might lead to a loss of richness in the data. However, composite competence measures are aimed to create a wholistic picture of the skills and are therefore suitable for the aim of this study. Additionally, the tests for math competence and motor competence are widely used and show high internal consistency (see Materials and methods section).

In sum, the present study aimed to explore the relationship between VSWM, LSK, math competence, motor competence, and potential gender differences in the relationships in first grade children. Overall, we argue that the current paper provides evidence for moderate relationships between skills in first grade which, as discussed earlier, partly contradicts previous findings. Based on the principles of Fleishman (1975) and Edelman (1993), one can argue that the moderate relationships represent a certain degree of skill specificity and that underlying cognitive capacities are influencing across the measured skill domains. We argue that the implications of the findings are that children should train with a high degree of specificity from first grade, e.g., by repeated stimuli with the right amount of intensity (Kleim and Jones, 2008), which in turn ensures a good foundation for basic cognitive skills at the start of school (Sigmundsson et al., 2017b). More research is needed to ascertain this argument. The lack of statistically significant gender differences in the relationships indicates that males and females are similar in first grade. As the research on gender differences in relationships between skills is lacking, the present study provides novel findings. Future research should examine this further. Some limitations were present, e.g., non-normal data and sample size, although the applicability of correlation analysis is well suited for the research question. Further studies could improve on some of these limitations by implementing larger sample sizes, as well as including more complex measures of math competence, reading, and VSWM. The special role of VSWM in early formal schooling ought to be explored further in future studies.

## Data availability statement

The raw data supporting the conclusions of this article will be made available by the authors, without undue reservation.

## Ethics statement

The studies involving human participants were reviewed and approved by Regional Committees for Medical and Health Research Ethics of Norway. Written informed consent to participate in this study was provided by the participants’ legal guardian/next of kin.

## Author contributions

AE, AO, and HS: idea, writing, and analyses. All authors contributed to the article and approved the submitted version.

## Conflict of interest

The authors declare that the research was conducted in the absence of any commercial or financial relationships that could be construed as a potential conflict of interest.

## Publisher’s note

All claims expressed in this article are solely those of the authors and do not necessarily represent those of their affiliated organizations, or those of the publisher, the editors and the reviewers. Any product that may be evaluated in this article, or claim that may be made by its manufacturer, is not guaranteed or endorsed by the publisher.
